# Higgs characterisation at NLO in QCD: CP properties of the top-quark Yukawa interaction

**DOI:** 10.1140/epjc/s10052-014-3065-2

**Published:** 2014-09-23

**Authors:** Federico Demartin, Fabio Maltoni, Kentarou Mawatari, Ben Page, Marco Zaro

**Affiliations:** 1 Centre for Cosmology, Particle Physics and Phenomenology (CP3), Université catholique de Louvain, 1348 Louvain-la-Neuve, Belgium; 2 Theoretische Natuurkunde and IIHE/ELEM, Vrije Universiteit Brussel, and International Solvay Institutes, Pleinlaan 2, 1050 Brussels, Belgium; 3 Departamento de Física Teórica y del Cosmos y CAFPE, Universidad de Granada, Granada, Spain; 4 Sorbonne Universités, UPMC University Paris 06, UMR 7589, LPTHE, 75005 Paris, France; 5 CNRS, UMR 7589, LPTHE, 75005 Paris, France

## Abstract

At the LHC the CP properties of the top-quark Yukawa interaction can be probed through Higgs production in gluon fusion or in association with top quarks. We consider the possibility for both CP-even and CP-odd couplings to the top quark to be present, and study CP-sensitive observables at next-to-leading order (NLO) in QCD, including parton-shower effects. We show that the inclusion of NLO corrections sizeably reduces the theoretical uncertainties, and confirm that di-jet correlations in $H+2$ jet production through gluon fusion and correlations of the top-quark decay products in $t\bar{t}H$ production can provide sensitive probes of the CP nature of the Higgs interactions.

## Introduction

The top-quark Yukawa interaction has played a crucial role in the recent discovery of the Higgs boson in the first run of the LHC [1–4]. It is thanks to its large value that production in gluon fusion (GF), which mostly proceeds through a top-quark loop in the Standard Model (SM), has provided the necessary statistics for discovery already with a modest integrated luminosity. The wealth of production and decay channels available for a SM scalar with a mass of about 125 GeV, has also made it possible to combine information from different final-state measurements [5]. Global coupling extractions [3, 6] provide indirect evidence that the Higgs boson couples to top quarks with a strength in agreement with the SM expectations. Furthermore, the first exploratory searches of associated Higgs production with a top-quark pair ($t\bar{t}H$), while not yet being sensitive enough for an observation, already set an upper bound on the strength of the interaction of 3–6 times the SM expectation [7–9]. In the coming run of the LHC at 13 TeV, $t\bar{t}H$ production will certainly serve as a key channel to test the SM and explore new physics.

While the path towards more and more precise determinations of the strength of the Yukawa interaction with the top (and of the Higgs boson couplings in general) is clear, the investigation of the structure and the properties of such interaction is considerably more open. One of the fundamental questions is whether the Higgs–top-quark coupling is CP violating, i.e. the Higgs couples to both scalar and pseudoscalar fermion densities. In this context, it is important to stress that so far all experimental determinations of the Higgs CP properties [4, 10] have been obtained from the $H\rightarrow VV\rightarrow 4\ell$ decay mode and therefore only constrain the $HVV$ interactions.

Gathering information on the CP properties of the top-quark Yukawa interaction is not an easy task. As there is no decay mode of the Higgs to or through top quarks that can be effectively studied at the LHC, only Higgs production can be considered. In addition, even though different couplings, either scalar, pseudoscalar or mixed, have an impact on the production rates [11–13] and can also be bound by indirect measurements [14], only specially designed observables can provide direct evidence of CP-violating effects at hadron colliders. In inclusive Higgs production, for example, at least two extra jets are needed in the final state to be able to construct CP-sensitive observables. These can probe the Higgs interaction with the top quark through GF [as well as with $W$ and $Z$’s in vector boson fusion (VBF)]. The $t\bar{t}H$ final state, on the other hand, provides many CP-sensitive observables that can also be constructed from the daughters of the top-quark decays. In fact, in $H+$ jets and $t\bar{t}H$ production information on the CP nature of the top-quark coupling is encoded in the correlations between the jets and among the top–antitop decay products. This means that the choice of decay mode of the Higgs in the corresponding analyses can be done freely and based on criteria other than the requirement of a precise reconstruction of the Higgs resonance, something that, in general, might not even be needed.

In order to test the different hypotheses for the Higgs sector, the approach based on an effective field theory (EFT) turns out to be particularly suitable, given the fact that the current experimental data do not show any clear sign of physics beyond the SM. In such an approach, no new particle and symmetry is hypothesised on top of the SM ones. This has the advantage of reducing the number of new parameters and interactions compared to other approaches based only on Lorentz symmetry, without losing the ability to describe in a model-independent way the effects of any new physics we cannot directly access at the current energies. Furthermore, the EFT approach can be systematically improved by including higher-dimensional operators in the lagrangian on the one hand (which are suppressed by higher powers of the scale $\Lambda$ where new physics appears), and higher-order perturbative corrections on the other hand.

The aim of this work is to present how EFT predictions accurate to next-to-leading order (NLO) in QCD matched to a parton shower can be used to determine the CP properties of the Higgs boson coupling to the top quark, through Higgs production in association with jets or with a pair of top quarks. To this aim we employ the *Higgs Characterisation* (HC) framework originally proposed in [15], which follows the general strategy outlined in [16] and has been recently applied to the VBF and VH channels [17]. In this respect, this work contributes to the general effort of providing NLO accurate tools and predictions to accomplish the most general and accurate characterisation of Higgs interactions in the main production modes at the LHC. Note that at variance with VBF and VH, $H+$ jets and $t\bar{t}H$ are processes mediated by QCD interactions at the Born level, hence higher-order corrections are expected to be more important and certainly needed in analyses aiming at accurate and precise extractions of the Higgs properties.

First, we consider Higgs production in GF together with extra jets, focusing on final states with at least two jets. This process is not only a background to VBF, but it can also provide complementary information on the Higgs boson coupling properties [18–24]. In the heavy-top limit, the CP structure of the Higgs–top interaction is inherited by the effective Higgs–gluon vertices [25–30]. Higgs plus two (three) jets through GF at LO has been computed in [31–34], where the full top-mass dependence was retained. The results cited above show that the large top-mass limit is a very good approximation as long as the transverse momentum of the jets is not sensibly larger than the top mass and justify the use of EFT approach for the Higgs–gluons interactions. In the $m_\mathrm{t}\rightarrow \infty$ limit, the resulting analytic expressions at NLO for GF $Hjj$ production have been implemented in MCFM [35], which has been used by Powheg Box [36] and Sherpa [37] to obtain NLO results matched with parton shower (NLO+PS). Independent NLO+PS predictions in the Sherpa package using GoSam [38] for the one-loop matrix elements and in MadGraph5_aMC@NLO [39], which embodies MadFKS [40] and MadLoop [41], are also available. We note that all the above predictions are for the SM Higgs boson, i.e. the CP-even state, and $Hjj$ production for the CP-odd state has been only available at LO, yet with the exact top-mass dependence [21]. In this paper we present NLO results in the large top-mass limit for GF production of a generic (mixed) scalar/pseudoscalar state in association with one or two jets at the LHC, also matching to parton shower.

Second, we study $t\bar{t}H$ production for arbitrary CP couplings, including NLO+PS effects. While NLO corrections in QCD for this process have been known for quite some time [42, 43], the NLO+PS prediction has been done only recently, for both CP eigenstates, $0^+$ and $0^-$, in aMC@NLO [44] and in the Powheg Box [45] for the CP-even case only. The spin-correlation effects of the top–antitop decay products have also been studied at the NLO+PS level with the help of MadSpin [46, 47]. Weak and electroweak corrections have also been reported recently in [48, 49], respectively. The phenomenology of a CP-mixed Higgs coupling to the top quark at the LHC has been studied at LO in [50]. In addition to the case where the Higgs has definite CP quantum numbers, here we consider the more general case of a CP-mixed particle ($0^\pm$) including NLO in QCD, parton-shower effects and spin-correlated decays.

The paper is organised as follows. In the next section we recall the effective lagrangian employed for a generic spin-0 resonance and define sample scenarios used to determine the CP properties of the Higgs boson. We also briefly describe our setup for the computation of NLO corrections in QCD together with matching to parton shower. In Sect. 3 we present results of $H+$ jets in GF, focusing on the $H+2$ jet production. We also make a comparison with VBF production with dedicated kinematical cuts. In Sect. 4 we illustrate the $t\bar{t}H$ production channel. In Sect. 5 we briefly summarise our findings and in Appendix we present the Feynman rules, the UV and the $R_2$ counterterms necessary to NLO computations for GF in the heavy-top-quark limit.

## Setup

In this section, we summarise our setup. We start from the definition of the effective lagrangian, pass to the identification of suitable benchmark scenarios, and finally to event generation at NLO in QCD accuracy, including parton-shower effects.

### Effective lagrangian and benchmark scenarios

The most robust approach to build an effective lagrangian is to employ all the SM symmetries, i.e. start from a linearly realised electroweak symmetry and systematically write all higher-dimensional operators, organised in terms of increasing dimensions. The complete basis at dimension six has been known for a long time [51, 52] and recently reconsidered in more detail in the context of the Higgs boson; see e.g., [53–55]. This approach has been followed in the FeynRules [56] implementation of [57], where the effective lagrangian is written in terms of fields above the electroweak symmetry breaking (EWSB) scale and then expressed in terms of gauge eigenstates.

In [15] we have followed an alternative approach (and yet fully equivalent in the context of the phenomenological applications of this paper, as explicitly seen in Tables 1 and 3 of [57]) and implemented the EFT lagrangian starting from the mass eigenstates, so below the EWSB scale, and for various spin–parity assignments ($X(J^P)$ with $J^P=0^{\pm },1^{\pm },2^+$). We have also used FeynRules, whose output in the UFO format [58, 59] can be directly passed to MadGraph5_aMC@NLO [39]. We stress that this procedure is fully automatic for computations at LO, while at NLO the UFO model has to be supplemented with suitable counterterms, as will be recalled in Sect. 2.2, a procedure that in this work has been performed by hand.

The term of interest in the effective lagrangian can be written as [see Eq. (2.2) in [15]]:

1 $$\begin{aligned} \mathcal{L}_0^t = -\bar{\psi }_\mathrm{t}\big ( c_{\alpha }\kappa _{\scriptscriptstyle Htt}g_{\scriptscriptstyle Htt} +i s_{\alpha }\kappa _{\scriptscriptstyle Att}g_{\scriptscriptstyle Att}\, \gamma _5 \big ) \psi _\mathrm{t}\, X_0, \end{aligned}$$

where $X_0$ labels the scalar boson, $c_{\alpha }\equiv \cos \alpha$ and $s_{\alpha }\equiv \sin \alpha$ can be thought of as “CP mixing” parameters, $\kappa _{\scriptscriptstyle Htt,Att}$ are the dimensionless real coupling parameters, and $g_{\scriptscriptstyle Htt}=g_{\scriptscriptstyle Att}=m_\mathrm{t}/v\,(=y_\mathrm{t}/\sqrt{2})$, with $v\sim 246$ GeV. While obviously redundant (only two independent real quantities are needed to parametrise the most general CP-violating interaction), this parametrisation has several practical advantages, among which the possibility of easily interpolating between the CP-even ($c_{\alpha }=1,s_{\alpha }=0$) and CP-odd ($c_{\alpha }=0,s_{\alpha }=1$) assignments as well as recovering the SM case by the dimensionless and dimensionful coupling parameters $\kappa _i$ and $g_{\scriptscriptstyle Xyy'}$.

The Higgs interaction with the top quarks induces a (non-decoupling) effective couplings to photons, gluons and photon-$Z$ gauge bosons through a top-quark loop. In the HC framework, the effective lagrangian for such loop-induced interactions with vector bosons reads [Eq. (2.4) in [15]]:

2 $$\begin{aligned}&\mathcal{L}_0^\mathrm{loop} =\bigg \{ -\frac{1}{4}\big [c_{\alpha }\kappa _{\scriptscriptstyle Hgg}g_{\scriptscriptstyle Hgg} \, G_{\mu \nu }^aG^{a,\mu \nu } \nonumber \\&\qquad \qquad +\,s_{\alpha }\kappa _{\scriptscriptstyle Agg}g_{\scriptscriptstyle Agg}\,G_{\mu \nu }^a\widetilde{G}^{a,\mu \nu } \big ] \nonumber \\&\qquad -\frac{1}{4}\big [c_{\alpha }\kappa _{\scriptscriptstyle H\gamma \gamma } g_{\scriptscriptstyle H\gamma \gamma } \, A_{\mu \nu }A^{\mu \nu } +s_{\alpha }\kappa _{\scriptscriptstyle A\gamma \gamma }g_{ \scriptscriptstyle A\gamma \gamma }\, A_{\mu \nu }\widetilde{A}^{\mu \nu } \big ] \nonumber \\&\qquad -\frac{1}{2}\big [c_{\alpha }\kappa _{\scriptscriptstyle HZ\gamma }g_{\scriptscriptstyle HZ\gamma } \, Z_{\mu \nu }A^{\mu \nu } +s_{\alpha }\kappa _{\scriptscriptstyle AZ\gamma }g_{\scriptscriptstyle AZ\gamma }\,Z_{\mu \nu }\widetilde{A}^{\mu \nu } \big ] \bigg \} X_0,\nonumber \\ \end{aligned}$$

where the (reduced) field strength tensors are defined as

3 $$\begin{aligned} G_{\mu \nu }^a&= \partial _{\mu }^{}G_{\nu }^a-\partial _{\nu }^{}G_{\mu }^a +g_sf^{abc}G_{\mu }^bG_{\nu }^c, \end{aligned}$$

4 $$\begin{aligned} V_{\mu \nu }&= \partial _{\mu }V_{\nu }-\partial _{\nu }V_{\mu }\quad (V=A,Z,W^{\pm }), \end{aligned}$$

and the dual tensor is

5 $$\begin{aligned} \widetilde{V}_{\mu \nu } =\frac{1}{2}\epsilon _{\mu \nu \rho \sigma }V^{\rho \sigma }. \end{aligned}$$

We note that the $X_0$–gluon lagrangian provides not only the $ggX_0$, but also the $gggX_0$ and $ggggX_0$ effective vertices; see the appendix.^1^ For the $X_0\gamma \gamma$ and $X_0Z\gamma$ interactions, in addition to the top-quark loop, a $W$-boson loop contributes for the CP-even case and in fact dominates. As a result, these processes are less sensitive to the CP properties of the top Yukawa coupling. The dimensionful loop-induced couplings $g_{\scriptscriptstyle Xyy'}$ are shown in Table 1. In the following, we focus only on the gluonic operators in Eq. (2). As mentioned in the introduction, the EFT prediction can be improved by including higher-dimensional operators, and this can be achieved rather easily in our framework by adding, e.g., the dimension-seven Higgs–gluon lagrangian [60] into the HC model. Finally, we recall that in the HC lagrangian the loop-induced $X_0ZZ$ and $X_0WW$ interactions are parametrised by the cutoff $\Lambda$, since those are sub-leading contributions to the SM tree-level interaction; see Eq. (6) below.

**Table 1:** Loop-induced couplings $g_{\scriptscriptstyle Xyy'}$ in the lagrangian (2). $c_W=\cos \theta _W$ and $C=\sqrt{\frac{\alpha _{\scriptscriptstyle \mathrm EM}G_F m_Z^2}{8\sqrt{2}\pi }}$

$g_{\scriptscriptstyle Xyy'}$	$gg$	$\gamma \gamma$	$Z\gamma$
$X=H$	$-\alpha _s/3\pi v$	$47\alpha _\mathrm{EM}/18\pi v$	$C (94 c^2_W-13)/9\pi v$
$X=A$	$\alpha _s/2\pi v$	$4\alpha _\mathrm{EM}/3\pi v$	$2 C (8c^2_W-5)/3\pi v$

In order to compare GF and VBF in the $Hjj$ channel, we also write the effective lagrangian for the interactions with massive gauge bosons (Eq. (2.4) in [15]):

6 $$\begin{aligned}&\mathcal{L}_0^{Z,W} =\left\{ c_{\alpha } \kappa _{\scriptscriptstyle \mathrm{SM}} \, \left[ \frac{1}{2}g_{\scriptscriptstyle HZZ}\, Z_\mu Z^\mu + g_{\scriptscriptstyle HWW}\, W^+_\mu W^{-\mu }\right] \right. \nonumber \\&\qquad \qquad \quad -\frac{1}{4}\frac{1}{\Lambda }\left[ c_{\alpha }\kappa _{\scriptscriptstyle HZZ} \, Z_{\mu \nu }Z^{\mu \nu } +s_{\alpha }\kappa _{\scriptscriptstyle AZZ}\,Z_{\mu \nu }\widetilde{Z}^{\mu \nu } \right] \nonumber \\&\quad -\frac{1}{2}\frac{1}{\Lambda }\left[ c_{\alpha }\kappa _{\scriptscriptstyle HWW} \, W^+_{\mu \nu }W^{-\mu \nu } +s_{\alpha }\kappa _{\scriptscriptstyle AWW}\,W^+_{\mu \nu }\widetilde{W}^{-\mu \nu }\right] \nonumber \\&\quad \left. -\frac{1}{\Lambda }c_{\alpha } \left[ \kappa _{\scriptscriptstyle H\partial Z} \, Z_{\nu }\partial _{\mu }Z^{\mu \nu } \!+\! \left( \kappa _{\scriptscriptstyle H\partial W} W_{\nu }^+\partial _{\mu }W^{-\mu \nu }\!+\!h.c.\right) \right] \right\} X_0, \nonumber \\ \end{aligned}$$

where $g_{\scriptscriptstyle HZZ}=2m_Z^2/v$ and $g_{\scriptscriptstyle HWW}=2m_W^2/v$ are the SM couplings, and $\Lambda$ is the cutoff scale. The HC model parameters are summarised in Table 2.

**Table 2:** HC model parameters

Parameter	Description
$\Lambda$ (GeV)	Cutoff scale
$c_{\alpha }$ ($\equiv$ $\cos \alpha$)	Mixing between $0^+$ and $0^-$
$\kappa _i$	Dimensionless coupling parameter

In Table 3 we list the representative scenarios that we later use for illustration. The first scenario, which we label $0^+$(SM), corresponds to the SM, with the couplings to fermions as described by Eq. (1), and the effective couplings to gluons as described by the corresponding gluonic operators in Eq. (2). The second scenario, which we label $0^-$, corresponds to a pure pseudoscalar state. The third scenario, $0^\pm$, describes a CP-mixed case, where the spin-0 boson is a scalar/pseudoscalar state in equal proportions.

**Table 3:** Benchmark scenarios for GF/$t\bar{t}H$

Scenario for GF/$t\bar{t}H$	HC parameter choice
$0^+$ (SM)	$\kappa _{\scriptscriptstyle Hgg/Htt}=1\ (c_{\alpha }=1)$
$0^-$	$\kappa _{\scriptscriptstyle Agg/Att}=1\ (c_{\alpha }=0)$
$0^{\pm }$	$\kappa _{\scriptscriptstyle Hgg,Agg/Htt,Att}=1\ (c_{\alpha }=1/\sqrt{2})$

To compare between $H+2$ jets in GF and in VBF, we collect in Table 4 some of the new physics scenarios considered in the previous HC paper [17]. The first scenario corresponds to the SM. The second scenario, $0^+$(HD), represents a scalar state interacting with the weak bosons in a custodial invariant way through the higher-dimensional (HD) operators of Eq. (6) corresponding to $\kappa _{\scriptscriptstyle HZZ, HWW}$. The third scenario, $0^-$(HD), is the analogous of a pure pseudoscalar state, while the fourth scenario is representative of a CP-mixed case, with equal contributions from the scalar and pseudoscalar components.

**Table 4:** Benchmark scenarios for VBF used for comparison with Higgs production in GF

Scenario for VBF	HC parameter choice
$0^+$ (SM)	$\kappa _{\scriptscriptstyle SM}=1\ (c_{\alpha }=1)$
$0^+$ (HD)	$\kappa _{\scriptscriptstyle HZZ,HWW}=1\ (c_{\alpha }=1)$
$0^-$ (HD)	$\kappa _{\scriptscriptstyle AZZ,AWW}=1\ (c_{\alpha }=0)$
$0^{\pm }$ (HD)	$\kappa _{\scriptscriptstyle HZZ,HWW,AZZ,AWW}=1\ (c_{\alpha }=1/\sqrt{2})$

### NLO corrections matched with parton shower

MadGraph5_aMC@NLO is designed to perform automatic computations of tree-level and NLO differential cross sections, including the possibility of matching LO and NLO calculations to parton showers via the MC@NLO method [61], and also to merge LO [62] and NLO [63] samples that differ in parton multiplicities. Currently, NLO computations are restricted to QCD corrections. They can be achieved fully automatically in the SM. Recently, the computation of ultraviolet (UV) and $R_2$ counterterms, the latter being originally necessary to compute one-loop amplitudes with the CutTools [64] implementation of the OPP integrand-reduction method [65], was automated for any renormalisable theory [66].

The UV and $R_2$ counterterms for QCD one-loop amplitudes in the SM were presented in [67] and have been available in MadGraph5_aMC@NLO for some time. The corresponding terms for effective interactions between the SM Higgs and gluons were presented in [68]. Here, we have derived them for the pseudoscalar case, listed in the appendix, and coded by hand in a UFO model named HC_NLO_X0. The resulting model is publicly available online in the FeynRules repository [69].

### Simulation parameters

We generate events for the LHC with centre-of-mass (CM) energies $\sqrt{s}=8$ and $13$ TeV, and we set the $X_0$ resonance mass to $m_{X_0}=125$ GeV. We take the heavy-top-quark limit for GF, while we set the top-quark mass to $m_{t}=173$ GeV in $t\bar{t}X_0$ production.

Parton distribution functions (PDFs) are evaluated by using the NNPDF2.3 (LO/NLO) parametrisation [70] through the LHAPDF interface [71]. For NLO predictions, the PDF uncertainty is computed together with the uncertainty in the strong coupling constant $\alpha _s(m_Z)$ as described in [72]. We assume the strong coupling constant to be distributed as a gaussian around the value

7 $$\begin{aligned} \alpha _s^{(\mathrm {NLO})}(m_Z) = 0.1190 \pm 0.0012~(68\,\%~\mathrm {C.L.}), \end{aligned}$$

where the confidence interval is taken accordingly to the PDF4LHC recommendation [73, 74]. At the present time there is no PDF set that allows the correct assessment of the PDF + $\alpha _s$ uncertainty at LO. Therefore, for LO predictions we compute the sole PDF uncertainty, with the strong coupling at the $m_Z$ scale fixed to $\alpha _s^{(\mathrm {LO})}(m_Z) = 0.130$ [75, 76].

Central values $\mu _0$ for the renormalisation and factorisation scales $\mu _{R,F}$ are set to

8 $$\begin{aligned} \mu _0^{(\mathrm {GF})} = H_\mathrm{T}/2 \end{aligned}$$

for $X_0$ (+jets) production in the GF channel,

9 $$\begin{aligned} \mu _0^{(\mathrm {VBF})} = m_W \end{aligned}$$

for $X_0jj$ production in the VBF channel, and

10 $$\begin{aligned} \mu _0^{(t\bar{t}H)} = \root 3 \of {m_\mathrm{T}(t)\,m_\mathrm{T}(\bar{t})\,m_\mathrm{T}(X_0)} \end{aligned}$$

for $t\bar{t}X_0$ production, where $m_\mathrm{T}\equiv \sqrt{m^2+p_\mathrm{T}^2}$ is the transverse mass of a particle, and $H_\mathrm{T}$ is the sum of the transverse masses of the particles in the final state. Uncertainties coming from missing higher orders are estimated varying $\mu _R$ and $\mu _F$, independently, by a factor 2 around $\mu _0$,

11 $$\begin{aligned} 1/2<\mu _{R,F}/\mu _\mathrm{0}<2. \end{aligned}$$

We note here that scale and PDF uncertainties are evaluated automatically at no extra computing cost via a reweighting technique [77]. In addition, such information is available on an event-by-event basis and therefore uncertainty bands can be plotted for any observables of interest. We define the total theoretical uncertainty of an observable as the linear sum of two terms: the PDF + $\alpha _s$ uncertainty on the one hand, and the overall scale dependence on the other.

For parton showering and hadronisation we employ HERWIG6 [78]. We recall that matching and merging to HERWIG++ [79], Pythia6 [80] (virtuality ordered, or $p_\mathrm{T}$ ordered for processes with no final-state radiation) and Pythia8 [81] are also available. Jets are reconstructed employing the anti-$k_\mathrm{T}$ algorithm [82] as implemented in FastJet [83], with distance parameter $R=0.4$ (both for jets in $H+$ jets production and for $b$-tagged jets coming from top decays in $t\bar{t}H$ production) and

12 $$\begin{aligned} p_\mathrm{T}(j)>30~\mathrm{GeV},\quad |\eta (j)|<4.5. \end{aligned}$$

## Gluon-fusion production with jets

In MadGraph5_aMC@NLO the code and the events for $X_0$ plus two jets in the GF channel can be automatically generated by issuing the following commands (note the / t syntax to forbid diagrams containing top loops): 
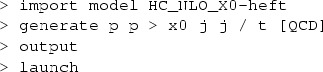
 where the -heft suffix in the model name refers to the corresponding model restriction. As a result, all the amplitudes featuring the Higgs–gluon effective vertices in the heavy-top limit are generated, including corrections up to NLO in QCD. Analogous commands can be issued to generate events for $X_0$ plus zero and one jet at NLO. The NLO computation for $Hjjj$ in GF has been recently achieved interfacing Sherpa with GoSam [84]. We note that MadGraph5_aMC@NLO provides the FxFx merging [63] to combine several NLO+PS samples, which differ by final-state multiplicities, and NLO merged Higgs production in GF was discussed in [39, 63].

As mentioned above, since our interest is geared towards QCD effects in production distributions, we do not include Higgs decays in our studies. We stress, however, that decays (as predicted in the HC model) can be efficiently included at the partonic event level by employing MadSpin [46], before passing the short-distance events to a parton-shower program.

### Total rates

We start by showing results for total cross sections for Higgs plus jet production in GF, not only for $H+2$ jets but also for $H+1$ jet as a reference. We remark here that as GF is the dominant Higgs production mechanism, enormous theoretical efforts to achieve more precise computation have been made over the last decade and we refer to the reports by the LHC Higgs Cross Section Working Group [85–87] for more details. We note that a first calculation of Higgs plus one jet at NNLO ($gg$ only and in the EFT) has been reported in [88].

Table 5 collects the LO and NLO total cross sections and the corresponding $K$ factors for $pp\rightarrow X_0 j$ at the 8- and 13-TeV LHC, together with uncertainties, for the three scenarios defined in Table 3. The acceptance cuts in Eq. (12) are imposed.

**Table 5:** LO and NLO cross sections and corresponding $K$ factors for $pp\rightarrow X_0+1$ jet (GF channel) at the 8- and 13-TeV LHC, for the three scenarios defined in Table 3. The integration error in the last digit(s) (in parentheses), and the fractional scale (left) and PDF (+$\alpha _s$) (right) uncertainties are also reported. In addition to fixed-order results, the PS-matched NLO cross sections and the ratios $R\equiv \sigma _\mathrm{NLO+PS}/\sigma _\mathrm{NLO}$ are also shown

Scenario	$\sigma _\mathrm{LO}$ (pb)	$\sigma _\mathrm{NLO}$ (pb)	$K$	$\sigma _\mathrm{NLO+PS}$ (pb)	$R$
LHC 8 TeV					
$0^+$	4.002(4)${}^{+46.8}_{-29.6}$ $\pm 3.3\,\%$	5.484(7)${}^{+17.0}_{-16.8}$ $\pm 1.2\,\%$	1.37	4.618${}^{+21.8}_{-18.8}$ $\pm 1.2\,\%$	0.84
$0^-$	9.009(9)${}^{+46.8}_{-29.6}$ $\pm 3.3\,\%$	12.34(2)${}^{+17.1}_{-16.8}$ $\pm 1.2\,\%$	1.37	10.38${}^{+21.7}_{-18.8}$ $\pm 1.2\,\%$	0.84
$0^\pm$	6.511(6)${}^{+46.8}_{-29.6}$ $\pm 3.3\,\%$	8.860(14)${}^{+16.9}_{-16.8}$ $\pm 1.2\,\%$	1.36	7.474${}^{+21.7}_{-18.8}$ $\pm 1.2\,\%$	0.84
LHC 13 TeV					
$0^+$	10.67(1)${}^{+41.7}_{-27.5}$ $\pm 2.6\,\%$	14.09(2)${}^{+16.2}_{-14.9}$ $\pm 1.1\,\%$	1.32	12.08${}^{+19.8}_{-16.7}$ $\pm 1.0\,\%$	0.86
$0^-$	24.01(2)${}^{+41.7}_{-27.5}$ $\pm 2.6\,\%$	31.67(6)${}^{+16.2}_{-14.9}$ $\pm 1.1\,\%$	1.32	27.14${}^{+20.3}_{-16.4}$ $\pm 1.0\,\%$	0.86
$0^\pm$	17.36(2)${}^{+41.7}_{-27.5}$ $\pm 2.6\,\%$	22.83(3)${}^{+16.2}_{-14.9}$ $\pm 1.1\,\%$	1.32	19.59${}^{+19.5}_{-16.6}$ $\pm 1.0\,\%$	0.86

Requiring the presence of jets in the final state entails imposing cuts at the generation level as well as after event generation in the case of NLO+PS simulation. We have checked that the cuts at the generation level were loose enough not to affect the NLO+PS rates and distributions. Since reconstructed jets after parton shower and hadronisation can be different from the fixed-order parton jets, the parton-shower matched cross section can be different from the fixed-order prediction.

The figure in parentheses is the integration error in the last digit(s). The first uncertainty (in percent) corresponds to the envelope obtained by varying independently the renormalisation and factorisation scales by a factor 2 around the central value, $\mu _0=H_\mathrm{T}/2$. The second one corresponds to the PDF ($+\alpha _s$) uncertainty. As mentioned in Sect. 2.3, the full PDF + $\alpha _s$ uncertainty is available only at NLO. It is well known that PDF and $\alpha _s$ uncertainties are comparable for GF at NLO [72], thus we take them both into account. We can see that both the scale dependence and the PDF + $\alpha _s$ uncertainties are independent of the scenarios, and as expected they are significantly reduced going from LO to NLO. It is also evident that the residual scale dependence is the dominant source of uncertainty in the GF channel. We also note that $\sigma (0^-)$ is larger than $\sigma (0^+)$ by a factor of 2.25 at LO (and to a good approximation even at NLO) due to the different coupling normalisation (see Table 1), and $\sigma (0^{\pm })$ is equal to the average of $\sigma (0^+)$ and $\sigma (0^-)$. This means that there are no interference effects in the total rates for this process.

In addition to the fixed-order results, we also show the NLO cross sections matched with parton shower ($\sigma _\mathrm{NLO+PS}$) in the table. The ratios to the fixed-order NLO rates, $R\equiv \sigma _\mathrm{NLO+PS}/\sigma _\mathrm{NLO}$ are shown in the last column. These ratios are smaller than one, as extra radiation generated by the parton shower tends to spread the energy of the original hard partons, affecting the spectrum of the jets and leading to more events which fail to pass the cuts. The survival rate after shower slightly increases as increasing the collision energy. We note that the ratios can slightly depend on the parton-shower programs [89], and these differences shall be considered as matching systematics. Another effect of the parton shower that we observe is a slightly increased scale dependence in the results, compared to the corresponding fixed-order predictions.

Table 6 presents results for $pp\rightarrow X_0+2$ jets. The features of the cross sections and uncertainties are qualitatively similar to the 1-jet case in Table 5, while rather different quantitatively. As one increases the number of extra jets, the cross section becomes smaller (as expected, yet mildly) and the $K$ factors are also reduced. On the other hand, the scale dependence increases, especially in the LO results, as more powers of $\alpha _s$ enter the matrix elements. Once again, the $K$ factors do not depend on the scenarios. We note that the LO ratio $\sigma (0^-)/\sigma (0^+)$ slightly deviates from 2.25 because of the missing $ggggA$ vertex as well as the different helicity structure of the amplitudes [90].

**Table 6:** Same as Table 5, but for $pp\rightarrow X_0+2$ jets (GF)

Scenario	$\sigma _\mathrm{LO}$ (pb)	$\sigma _\mathrm{NLO}$ (pb)	$K$	$\sigma _\mathrm{NLO+PS}$ (pb)	$R$
LHC 8 TeV					
$0^+$	1.351(1)${}^{+67.1}_{-36.8}$ $\pm 4.3\,\%$	1.702(6)${}^{+19.7}_{-20.8}$ $\pm 1.7\,\%$	1.26	1.276${}^{+29.4}_{-23.9}$ $\pm 1.7\,\%$	0.75
$0^-$	2.951(3)${}^{+67.2}_{-36.8}$ $\pm 4.4\,\%$	3.660(15)${}^{+19.1}_{-20.6}$ $\pm 1.7\,\%$	1.24	2.755${}^{+29.8}_{-24.1}$ $\pm 1.8\,\%$	0.75
$0^\pm$	2.142(2)${}^{+67.1}_{-36.8}$ $\pm 4.4\,\%$	2.687(10)${}^{+19.6}_{-20.8}$ $\pm 1.7\,\%$	1.25	2.022${}^{+29.7}_{-24.1}$ $\pm 1.8\,\%$	0.75
LHC 13 TeV					
$0^+$	4.265(4)${}^{+61.5}_{-34.9}$ $\pm 3.3\,\%$	5.092(23)${}^{+15.4}_{-17.9}$ $\pm 1.2\,\%$	1.19	4.025${}^{+23.9}_{-21.3}$ $\pm 1.2\,\%$	0.79
$0^-$	9.304(9)${}^{+61.6}_{-34.9}$ $\pm 3.4\,\%$	11.29(4)${}^{+16.0}_{-18.2}$ $\pm 1.2\,\%$	1.21	8.701${}^{+24.6}_{-21.6}$ $\pm 1.3\,\%$	0.77
$0^\pm$	6.775(6)${}^{+61.5}_{-34.9}$ $\pm 3.3\,\%$	8.055(35)${}^{+15.8}_{-18.2}$ $\pm 1.2\,\%$	1.19	6.414${}^{+24.4}_{-21.5}$ $\pm 1.2\,\%$	0.80

### Distributions

In the previous section we have seen that if the strength of the scalar and pseudoscalar couplings in the Higgs–top-quark interaction is similar [i.e. $\kappa _{\scriptscriptstyle Htt}g_{\scriptscriptstyle Htt}\sim \kappa _{\scriptscriptstyle Att}g_{\scriptscriptstyle Att}$ in Eq. (1)], the total Higgs production rate in GF is sensitive to the CP mixing of the Higgs boson. We now turn to distributions, where GF jet–jet correlations are known tools to determine the Higgs CP properties [18–24]. In the following, all the distributions will be shown for the 13-TeV LHC. For these studies, we require the presence of at least two reconstructed jets in the final states. The jets are ordered by the transverse momenta.

**Fig. 1:**
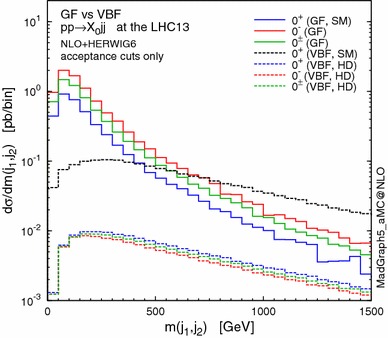
Distribution of the invariant mass of the two leading jets in $pp\rightarrow X_0jj$ through GF (*solid lines*) and VBF (*dashed*) at the 13-TeV LHC. The different hypotheses are defined in Tables 3 and 4

We start by showing the invariant mass distribution $m_{jj}$ of the two leading jets in Fig. 1, where GF and VBF are compared for the various scenarios defined in Tables 3 and 4. For the VBF HD scenarios we fix the cutoff scale to $\Lambda =1$ TeV. GF is dominant in the small di-jet mass region, while VBF tends to produce a jet pair with higher invariant mass [32]. This is because, for $Hjj$ production in GF, the $gg$ and $qg$ initial states are dominant, and hence the Higgs can be radiated off the initial or final gluon legs, leading to more central jets with the acceptance cuts only. For the VBF process, on the other hand, the Higgs boson is produced through the $t,u$-channel weak-boson fusion, leading to forward hard jets. Based on this fact, we usually require a minimum $m_{jj}$ as a VBF cut in order to minimise the GF contribution to extract the VBF information. The shapes of the $m_{jj}$ spectra are similar among the different CP scenarios within the same channel. This means that, apart from the difference between GF and VBF, the invariant mass cut acts in a similar way on every CP scenario in a given channel; more details for the VBF case can be found in [17].

Looking at the subprocesses contributing to $X_0+2$ jets is instructive. The $qq \rightarrow X_0 qq$ subprocess features VBF-like $t$-channel gluon exchange diagram and is not affected by the $m_{jj}$ cut, since the jets tend to be produced in the forward region, similarly to the weak-boson case [23]. Moreover, even for the $gg$ and $qg$ induced subprocesses, the $t$-channel contribution becomes relatively important by imposing the invariant mass cut. In other words, the VBF cut maximises the contributions featuring gluons in the $t$-channel, which are the most sensitive to the CP properties of $X_0$ also in the GF case [19]. To illustrate how the CP-sensitive observables change with the VBF cut, on top of the acceptance cuts, we impose an invariant mass cut as

13 $$\begin{aligned} m(j_1,j_2)>250,\ 500~\mathrm{GeV}. \end{aligned}$$

We do not require a minimum rapidity separation, although this is another common VBF cut, since $\Delta \eta _{jj}$ itself is an observable sensitive to the CP properties of $X_0$ [23, 91].

**Fig. 2:**
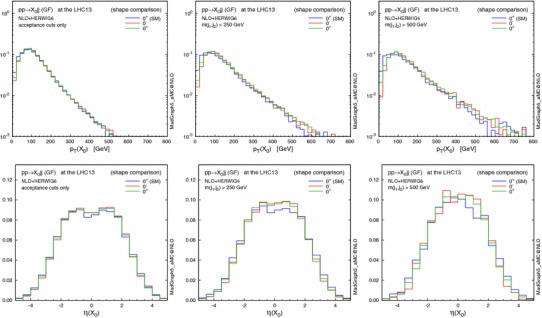
Normalised distributions (shape comparison) in $p_\mathrm{T}$ and $\eta$ of the resonance $X_0$, with the acceptance cuts for jets (*left*), plus $m(j_1,j_2)>250$ GeV (*centre*) and $500$ GeV (*right*). The three spin-0 hypotheses are defined in Table 3

**Fig. 3:**
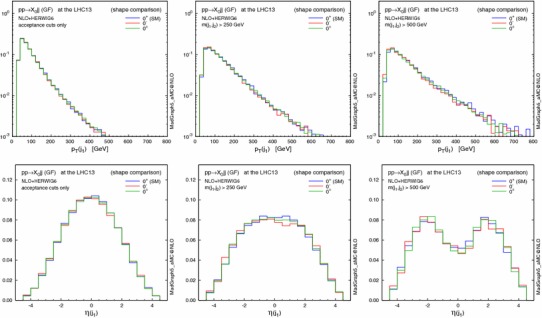
Same as Fig. 2, but for the leading jet

Figures 2 and 3 show the effect of the invariant mass cut on the $p_\mathrm{T}$ and $\eta$ distributions for the resonance $X_0$ and the leading jet. Imposing larger $m_{jj}$ cuts leads to harder transverse momenta for both the $X_0$ and the jets; as a result, the $X_0$ is produced more centrally, while the jets are shifted to the forward regions and the difference in the low $p_\mathrm{T}(X_0)$ region between the various CP scenarios becomes more pronounced. This behaviour is due to the fact that at larger $m_{jj}$ topologies featuring the emission of the Higgs boson by a gluon exchanged in the $t$-channel are enhanced, similarly to the typical VBF topology.

A possible concern is to what extent the EFT approach is valid. In fact the heavy-top-quark effective lagrangian in Eq. (2) is a good approximation for single light-Higgs production. The EFT closely reproduces the $m_{jj}$ spectrum of the loop computation even in the very high invariant mass region [32]. However, this approximation fails when the transverse momenta of the jets are larger than the top mass [31], overestimating the exact prediction for the $p_\mathrm{T}(j_{1})>m_\mathrm{t}$ region. Since the events are generated predominantly in the small $p_\mathrm{T}(j_1)$ region, we choose not to apply any rejection of events with large $p_\mathrm{T}$ in the following analyses.

**Fig. 4:**
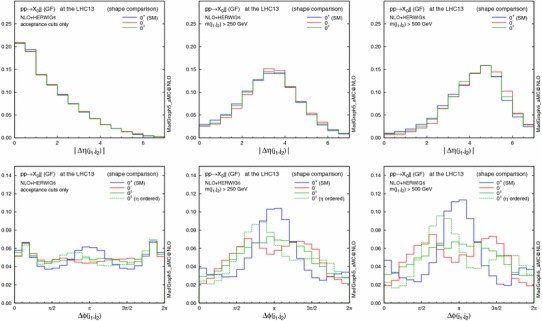
Same as Fig. 2, but for $\Delta \eta$ and $\Delta \phi$ distributions between the two tagging jets. For $\Delta \phi$, the distribution with the additional $\eta$ jet ordering is also shown by a *dashed line* for the $0^{\pm }$ case

**Fig. 5:**
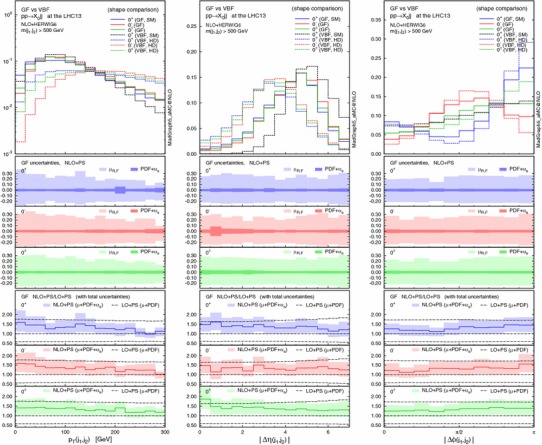
Normalised distributions (shape comparison) of the $p_\mathrm{T}$ of the di-jet system (*left*), the rapidity (*centre*) and azimuthal (*right*) difference between the two tagging jets for $pp\rightarrow X_0jj$ in GF (*solid lines*) and VBF (*dashed*) at the 13-TeV LHC, where the acceptance cuts plus the $m_{jj}>500$ GeV cut are applied. For each GF scenario, the *middle panels* show the scale and PDF + $\alpha _s$ uncertainties, while the *bottom ones* give the ratio of NLO+PS to LO+PS results with the total uncertainties

The most sensitive observables for the CP nature of the Higgs boson couplings to the top quark in this channel are di-jet correlations, shown in Fig. 4. As already seen in Fig. 3, the invariant mass cut effectively suppresses the central jet activity, although the different CP scenarios in the rapidity separation $\Delta \eta _{jj}\equiv \eta (j_1)-\eta (j_2)$ can be hardly distinguished. On the other hand, the azimuthal angle between the two jets $\Delta \phi _{jj}\equiv \phi (j_1)-\phi (j_2)$ is well known to be very sensitive to the CP mixing and our results confirm that this is indeed the case also at NLO (for a LO vs. NLO comparison see Fig. 5 in the following).

A remarkable observation is that the $\Delta \phi _{jj}$ distribution is more sensitive to the CP-mixed state, when the two leading jets (ordered by $p_\mathrm{T}$) are reordered in pseudorapidity^2^ (dashed green), compared to the distribution with the usual $p_\mathrm{T}$ jet ordering (solid green). This is especially true for the maximal mixing scenario, which we consider here, since with just $p_\mathrm{T}$ ordering the $\pi /4$ phase shift, generated by quantum interference between the CP-even and -odd components, is cancelled between $+\Delta \phi _{jj}$ and $-\Delta \phi _{jj}$ [18]. Indeed, the distribution for $0^{\pm }$ without $\eta$ ordering is just the weighted average of the $0^+$ and $0^-$ cases.

The NLO computation allows also to investigate the effect of applying a veto on additional jets in the event, a procedure that is known to suppress the central QCD activities and to enhance the VBF signal [92, 93]. We implement it by vetoing events containing a third jet laying in pseudorapidity between the forward and backward tagging jets,

14 $$\begin{aligned} \min \big \{ \eta (j_1), \eta (j_2) \big \} < \eta (j_\mathrm{veto}) < \max \big \{ \eta (j_1), \eta (j_2) \big \}. \end{aligned}$$

Table 7 collects the selection efficiencies on the NLO+PS samples after $m_{jj}>250$ and 500 GeV cuts, and $m_{jj}>500$ GeV plus the central jet veto, with respect to the acceptance cuts only. As already seen in Fig. 1, the efficiencies are very similar among the different scenarios. The additional jet veto could be useful to enhance the sensitivity to CP mixing, especially for the 13-TeV run. Indeed we have checked that the size of the variation in the $\Delta \phi _{jj}$ distribution in Fig. 4 becomes slightly larger. The related jet binning uncertainties have been discussed in detail in [94].

Finally, we discuss the theoretical uncertainties for the CP-sensitive observables. Figure 5 displays, from left to right, normalised distributions of the $p_\mathrm{T}$ of the di-jet system [which is equivalent to $p_\mathrm{T}(X_0)$ only at LO], the pseudorapidity and the azimuthal difference between the two tagging jets for $pp\rightarrow X_0+2$ jets in GF (solid lines) at the 13-TeV LHC. The acceptance cuts and the invariant mass cut $m_{jj}>500$ GeV are imposed. The middle panels show the scale and PDF + $\alpha _s$ uncertainties for each scenario, while the bottom ones give the ratio of NLO+PS to LO+PS results with the total theoretical uncertainties. The total uncertainty is defined as the linear sum of the scale and PDF + $\alpha _s$ uncertainties. The scale uncertainty is dominant, as observed in Table 6, and both the scale and PDF + $\alpha _s$ uncertainties change very mildly over the phase space. In all cases NLO corrections are relevant and cannot be described by an overall $K$ factor.

**Table 7:** Selection efficiencies with different di-jet invariant mass cuts for $pp\rightarrow X_0jj$. A jet veto defined in (14) is also applied in the last column

$m_{jj}>$	250 GeV (%)	500 GeV (%)	500 GeV + jet veto (%)
LHC 8 TeV			
$0^+$	22.7	6.6	5.0
$0^-$	21.4	5.7	4.5
$0^\pm$	21.5	6.2	4.6
LHC 13 TeV			
$0^+$	26.3	9.0	6.4
$0^-$	25.4	8.6	6.2
$0^\pm$	25.6	8.6	6.2

In the main panel, we also draw a comparison with the VBF contributions (dashed lines). The $p_\mathrm{T}(j_1,j_2)$ and $\Delta \eta (j_1,j_2)$ distributions show that in the SM VBF case the Higgs boson is produced more centrally while the tagging jets are more forward than in GF production. For the three HD VBF cases, conversely, the jets are more central. We recall that the type of operators are the same both for the GF and the HD VBF, i.e. the dimension-five operators $X_0V_{\mu \nu }V^{\mu \nu }$ and $X_0V_{\mu \nu }\widetilde{V}^{\mu \nu }$.

We track down the slight difference between GF and HD VBF in $\Delta \eta _{jj}$ to the presence of the mass of the $t$-channel vector boson, i.e. massless gluons vs. massive weak bosons. On the other hand, the slightly weaker modulation for $\Delta \phi _{jj}$ in GF is due to the presence of the $gg$ and $qg$ initiated contributions [19, 23]. We note that the interference between GF and VBF can be safely neglected [95, 96].

## Associated production with a top-quark pair

The code and events for $t\bar{t}X_0$ hadroproduction can be automatically generated by issuing the following commands in MadGraph5_aMC@NLO: 
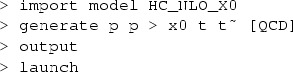


The top-quark decays are subsequently performed starting from the event file (in the Les Houches format [97]) by MadSpin [46] following a procedure [98] that keeps production and decay spin correlations intact.

### Total rates

In Table 8 we show results for total cross sections at LO and NLO accuracy and the corresponding $K$ factors at 8- and 13-TeV LHC for the three scenarios defined in Table 3. The uncertainties correspond respectively to (1) the integration error on the last digit(s), reported in parentheses, (2) the envelope obtained by independently varying the renormalisation and factorisation scales by a factor 2 around the central value given in Eq. (10), and (3) the PDF + $\alpha _s$ uncertainty (only PDF uncertainty for LO).

**Table 8:** LO and NLO cross sections and corresponding $K$ factors for $pp\rightarrow t\bar{t}X_0$ at the 8- and 13-TeV LHC, for the three scenarios defined in Table 3. The integration error in the last digit(s) (in parentheses), and the fractional scale (left) and PDF (+$\alpha _s$) (right) uncertainties are also reported. In addition to the fixed-order results, the PS-matched NLO cross sections for the di-leptonic decay channel $\sigma _\mathrm{NLO+PS}^\mathrm{dilep}$ and the ratios $R\equiv \sigma _\mathrm{NLO+PS}^\mathrm{dilep}/\sigma _\mathrm{NLO}$ are also shown, where the acceptance cuts in Eqs. (15) and (16) are applied

Scenario	$\sigma _\mathrm{LO}$ (fb)	$\sigma _\mathrm{NLO}$ (fb)	$K$	$\sigma _\mathrm{NLO+PS}^\mathrm{dilep}$ (fb)	$R$
LHC 8 TeV					
$0^+$	130.3(1)${}^{+36.8}_{-24.6}$ $\pm 5.9\,\%$	134.9(2)${}^{+3.2}_{-8.3}$ $\pm 3.0\,\%$	1.04	3.088${}^{+3.1}_{-8.4}$ $\pm 2.8\,\%$	$2.29 \times 10^{-2}$
$0^-$	44.49(4)${}^{+42.5}_{-27.6}$ $\pm 10.3\,\%$	47.07(6)${}^{+6.5}_{-11.5}$ $\pm 4.9\,\%$	1.06	1.019${}^{+5.5}_{-11.0}$ $\pm 4.3\,\%$	$2.16 \times 10^{-2}$
$0^\pm$	87.44(8)${}^{+38.2}_{-25.4}$ $\pm 6.9\,\%$	90.93(12)${}^{+3.9}_{-9.1}$ $\pm 3.4\,\%$	1.04	2.052${}^{+3.6}_{-9.0}$ $\pm 3.2\,\%$	$2.26 \times 10^{-2}$
LHC 13 TeV					
$0^+$	468.6(4)${}^{+32.8}_{-22.8}$ $\pm 4.5\,\%$	525.1(7)${}^{+5.7}_{-8.7}$ $\pm 2.1\,\%$	1.12	11.52${}^{+5.5}_{-8.7}$ $\pm 2.0\,\%$	$2.19 \times 10^{-2}$
$0^-$	196.8(2)${}^{+37.1}_{-25.2}$ $\pm 7.5\,\%$	224.3(3)${}^{+6.8}_{-10.5}$ $\pm 3.2\,\%$	1.14	4.488${}^{+5.6}_{-9.8}$ $\pm 2.8\,\%$	$2.00 \times 10^{-2}$
$0^\pm$	332.4(3)${}^{+34.0}_{-23.5}$ $\pm 5.4\,\%$	374.1(5)${}^{+6.0}_{-9.3}$ $\pm 2.5\,\%$	1.13	8.022${}^{+5.4}_{-8.9}$ $\pm 2.2\,\%$	$2.14 \times 10^{-2}$

At variance with the GF process, the production rate for the pseudoscalar case is smaller than that for the scalar case. Such a difference is proportional to the top-quark mass, as the amplitudes for the scalar and pseudoscalar interactions are identical in the limit where the Yukawa coupling is kept constant and the quark mass is neglected. In $pp$ collisions at the LHC energies the contribution of the $gg$ initial state is dominant over $q\bar{q}$ annihilation for all the scenarios. It is rather interesting to observe, however, that for a CP-odd scalar $q\bar{q}$ annihilation contributes at LO to just 16 % (10 %) of the total cross section at 8 (13) TeV, compared to around 40 % (30 %) of the SM-like CP-even case. This difference is such that the CP-odd case exhibits slightly larger scale and PDF uncertainties. Once again, we note that the scale dependence is larger than the PDF + $\alpha _s$ uncertainty (though not by as much as in GF $H+$ jets), and that all the uncertainties are significantly reduced going from LO to NLO, as expected. Increasing the collision energy from 8 to 13 TeV enhances the cross sections by about a factor 4, while the $K$ factors only slightly increase. As in the GF case, $\sigma (0^{\pm })$ is equal to the average of $\sigma (0^+)$ and $\sigma (0^-)$. We have verified explicitly that at the LO the interference between amplitudes corresponding to different parity interactions is exactly zero. At NLO, the interference at the amplitude level is nonzero, yet the total rates do sum up to each of the parity-definite contributions.

To investigate the spin correlations effects among the decay products from the top and antitop quarks, we present results for the di-leptonic decay channel of the top pair, $t\rightarrow b\ell ^+\nu _\ell$ and $\bar{t}\rightarrow \bar{b}\ell ^-\bar{\nu }_\ell$ with $\ell =e,\mu$. We require two leptons and two $b$-tagged jets that pass the acceptance cuts, respectively,

15 $$\begin{aligned} p_\mathrm{T}(\ell )>20~\mathrm{GeV},\quad |\eta (\ell )|<2.5, \end{aligned}$$

and

16 $$\begin{aligned} p_\mathrm{T}(j_b)>30~\mathrm{GeV},\quad |\eta (j_b)|<2.5. \end{aligned}$$

It is well known that dedicated top and Higgs reconstruction are crucial in order to obtain the significant $t\bar{t}H$ signal over the background, at least for the dominant $H\rightarrow b\bar{b}$ decay channel. Several proposals have been put forward from using multivariate analysis, e.g., matrix element method [99] to jet substructure/boosted techniques [100–103]. In this work we are mainly concerned in checking what observables can be sensitive to CP effects and do not consider either backgrounds or reconstruction issues. However, we will consider how CP-sensitive observables are affected by the requirement of a large transverse momentum for the Higgs, i.e. a “boosted Higgs”.

In Table 8, we also report the PS-matched NLO cross sections for the di-leptonic decay channel and the corresponding ratios to the fixed-order NLO prediction, $R\equiv \sigma _\mathrm{NLO+PS}^\mathrm{dilep}/\sigma _\mathrm{NLO}$, where acceptance cuts (assuming 100 % $b$-tag and lepton efficiencies) are taken into account. Accounting for the branching fraction of the di-lepton mode, $(0.213)^2\sim 0.045$, the ratios show that parton shower and the cuts lead to a decrease of about a factor 2 in the cross section. Increasing the CM energy results in the slightly smaller $R$ ratios.

### Distributions

**Fig. 6:**
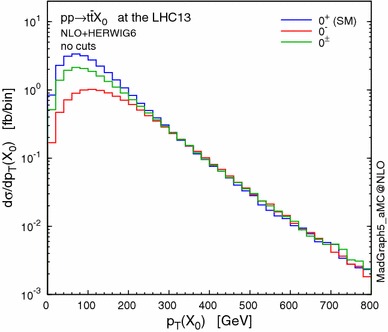
Distribution of the transverse momentum of $X_0$ in $pp\rightarrow t\bar{t}X_0$ at the 13-TeV LHC. The different hypotheses are defined in Table 3

In Fig. 6 we show differential cross sections for $t\bar{t}X_0$ production at the 13-TeV LHC as a function of the transverse momentum of the resonance $p_\mathrm{T}(X_0)$. As one can see, the difference between the various scenarios is significant in the low-$p_\mathrm{T}$ region, while the high-$p_\mathrm{T}$ tail of the distributions, featuring exactly the same shape, is not sensitive to the CP mixing [44]. It is also interesting to see that our normalisation choice, $g_{\scriptscriptstyle Htt}=g_{\scriptscriptstyle Att}=m_\mathrm{t}/v\,(=y_\mathrm{t}/\sqrt{2})$ leads to exactly the same rates at high $p_\mathrm{T}$ independently of the mixing parameter $\alpha$. This is a known feature of scalar radiation from a heavy quark at high $p_\mathrm{T}$ [42, 104, 105]. This raises the important question whether boosted analyses can be sensitive to CP properties of the Higgs–top-quark coupling, which we address below.

**Fig. 7:**
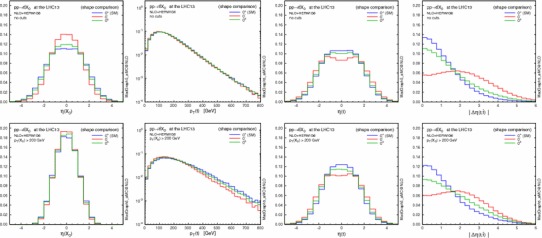
Normalised distributions (shape comparison) without cuts (*top*), while with the $p_{T}(X_0)>200$ GeV cut (*bottom*). The three spin-0 hypotheses are defined in Table 3

Figure 7 shows some other relevant distributions in the $t \bar{t} X_0$ final state, without and with the $p_\mathrm{T}(X_0)>200$ GeV cut: the pseudorapidity distribution of $X_0$, the top-quark transverse momentum and pseudorapidity, and the pseudorapidity distance between the top and antitop quarks $\Delta \eta (t,\bar{t})\equiv \eta (t)-\eta (\bar{t})$. Compared to the SM, a CP-odd $X_0$ tends to be produced more centrally, while the accompanying top quarks are more forward. The most sensitive distribution to CP mixing is the rapidity difference between the top and antitop. This observable is hardly affected by the $p_\mathrm{T}(X_0)>200$ GeV cut, thus the correlations among the top–antitop decay products provide a good CP-discriminating power also in the boosted regime.

**Fig. 8:**
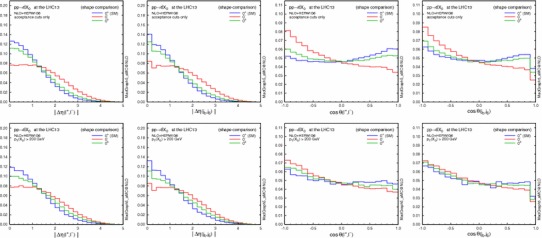
Normalised distributions (shape comparison) for the correlations between the top-quark decay products with the acceptance cuts (*top*) plus the $p_{T}(X_0)>200$ GeV cut (*bottom*)

In Fig. 8, we show the correlations between the top decay products (in the di-leptonic channel). As expected from the $\Delta \eta _{t\bar{t}}$ distribution, $\Delta \eta _{\ell \bar{\ell }}$ and $\Delta \eta _{b\bar{b}}$  are almost insensitive to the $p_\mathrm{T}(X_0)$ cut, while the angles between the leptons and between the $b$-jets are significantly affected by the boost. The angular observables in different frames have been studied in [47]. We note that, although we only consider the fully leptonic channel here, there is no limitation to study the semi-leptonic and fully hadronic channels by using MadSpin.

**Fig. 9:**
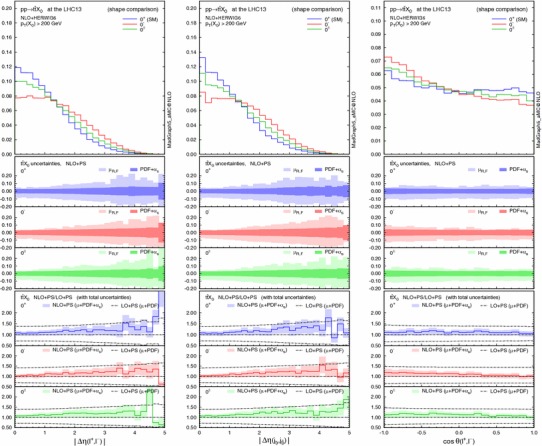
Normalised distributions (shape comparison) of the rapidity separation between the leptons (*left*) and the $b$-jets (*centre*), and the opening angle between the leptons (*right*), for $pp\rightarrow t\bar{t}X_0$ at the 13-TeV LHC, where the acceptance cuts plus $p_\mathrm{T}(X_0)>200$ GeV are applied. For each scenario, the *middle panels* show the scale and PDF + $\alpha _s$ uncertainties, while the *bottom ones* give the ratio of NLO+PS to LO+PS results, with total uncertainties

Finally, we discuss the theoretical uncertainties. Figure 9 displays, from left to right, the rapidity distance between the leptons ($\Delta \eta _{\ell \bar{\ell }}$) and between the $b$-tagged jets ($\Delta \eta _{b\bar{b}}$), and the opening angle between the leptons ($\cos \theta _{\ell \bar{\ell }}$), where the acceptance cuts in Eqs. (15) and (16) plus the $p_\mathrm{T}(X_0)>200$ GeV cut are applied. The middle panels show the uncertainties due to the scale variation and the PDF + $\alpha _s$ for each scenario, while the bottom ones give the ratio of NLO+PS to LO+PS results, each one with its total uncertainty band. We can see that, depending on the observable considered, the NLO corrections and the corresponding uncertainties can change significantly over the phase space. As in the $H$ + jets case, NLO corrections are significant for all the observables, considerably reduce the theoretical uncertainty, and cannot be described by an overall $K$ factor.

## Summary

In this work we have presented for the first time results at NLO in QCD, including parton-shower effects, for the hadroproduction of a spin-0 particle with CP-mixed coupling to the top quark, in gluon-fusion plus one and two jets and in association with a top-quark pair. Our results are obtained in a fully automatic way through the implementation of the relevant interactions in FeynRules and then performing event generation in the MadGraph5_aMC@NLO framework.

We have presented illustrative distributions obtained by interfacing NLO parton-level events to the HERWIG6 parton shower. NLO corrections improve the predictions of total cross sections by reducing PDF + $\alpha _s$ uncertainty and scale dependence. In addition, our simulations show that NLO+PS effects need to be accounted for to make accurate predictions on the kinematical distributions of the final-state objects, such as the Higgs boson, the jets and the top decay products.

We have confirmed that di-jet correlations in Higgs plus two jet production, in particular the azimuthal difference between the jets, are sensitive probes of the CP mixing of the Higgs. In associated production with a top pair, we have shown that many correlations between the top and antitop decay products can be sensitive to the CP nature of the Higgs. In particular, the pseudorapidity separation between the leptons or between the $b$-jets is a promising observable when analysing events with a Higgs boson at high transverse momentum. The quantitative determination of the CP mixing has been done for the GF channel at LO in [24], while the LO parton-level analysis has been done for the $t\bar{t}H$ channel including $tH$ and $\bar{t}H$ in [50]. The estimation of the impact of the NLO+PS corrections as well as detector effects is desired and will be reported elsewhere.

As a final remark, we note that in this work we have only addressed the issue of the CP properties of the flavour-diagonal Higgs–top-quark interactions, which can be parametrised in full generality as in Eq. (1). At the dimension-six level, however, other operators appear that lead to effective three-point and four-point Higgs–top-quark interactions of different type [106–110], including flavour changing neutral ones [106, 111, 112], which can also be studied in the same production channels as discussed here, i.e. $H$ + jets and $t\bar{t} H$. Work in promoting predictions for these processes to NLO accuracy in QCD is in progress.

## Figures and Tables

**Table 9:**
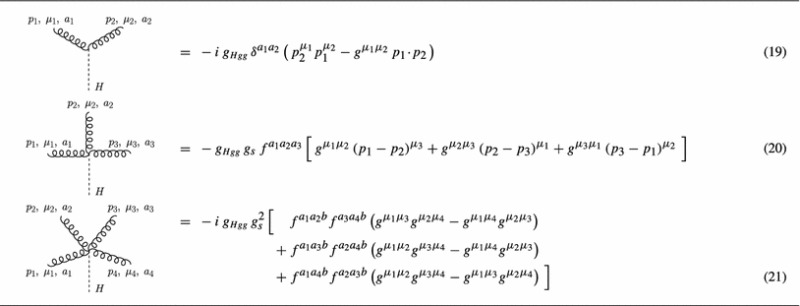
Feynman rules derived from the lagrangian (17)

**Table 10:**
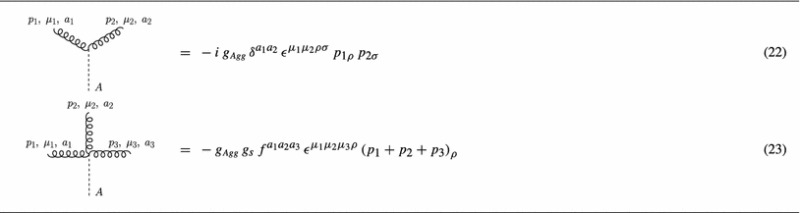
Feynman rules derived from the lagrangian (18). Note that all the pseudoscalar amplitudes vanish when $p_A^\mu \rightarrow (0,\mathbf {0})$

**Table 11:**
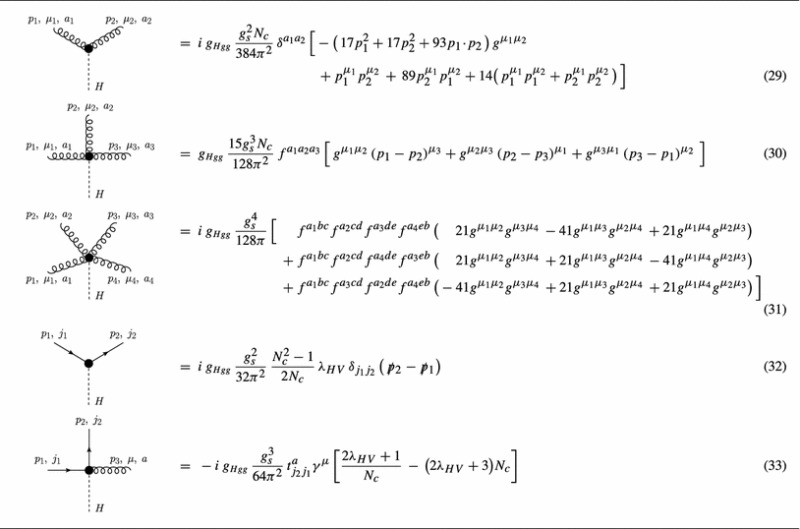
$R_2$ counterterms for the lagrangian (17). $\lambda _{HV}=1$ is for dimensional regularisation, while $\lambda _{HV}=0$ for dimensional reduction

**Table 12:**
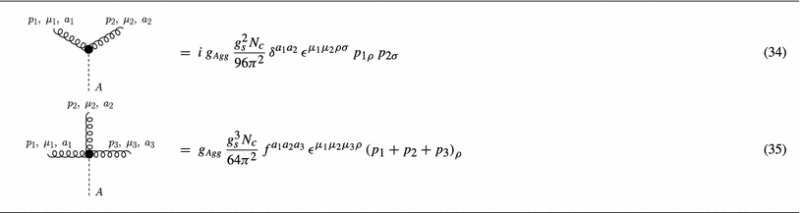
$R_2$ counterterms for the lagrangian (18)

## Appendix: Feynman rules, UV and $\varvec{R}_2$ terms for gluon-fusion Higgs production at NLO QCD

In this appendix we present the Feynman rules, UV and $R_2$ terms necessary for NLO-QCD automatic computations, for gluon fusion in an EFT approach, where the Higgs boson couples to gluons through loops of infinitely heavy quarks. The LO rules have been obtained automatically by coding the effective lagrangian in FeynRules, while the UV and $R_2$ terms have been coded by hand in the UFO format. This file is read by ALOHA [59], which generates a library of helicity amplitudes and currents for a given process as requested by the user in MadGraph5_aMC@NLO.

In this note, we use the following conventions: outgoing momenta for external particles; the antisymmetric tensor $\epsilon ^{0123}=+1$; and the metric tensor $g^{\mu \nu }=\text {diag}(1,-1,-1,-1)$.

The relevant Higgs–gluon interaction lagrangian consists of the first two operators in Eq. (2). Since it is linear in the scalar and pseudoscalar components of $X_0$, we only need to consider the two separate cases of a pure scalar $X_0=H$ [i.e. $c_\alpha =1,\,\kappa _{\scriptscriptstyle Hgg}\ne 0$ in Eq. (6)], or a pure pseudoscalar $X_0=A$ (i.e. $s_\alpha =1,\,\kappa _{\scriptscriptstyle Agg}\ne 0$). Thus, we start from the two effective lagrangians

17 $$\begin{aligned} \mathcal {L}_{H}&= -\frac{1}{4}\, g_{\scriptscriptstyle Hgg}\, G_{\mu \nu }^a G^{a,\mu \nu } H, \end{aligned}$$

18 $$\begin{aligned} \mathcal {L}_{A}&= -\frac{1}{4}\, g_{\scriptscriptstyle Agg}\, G_{\mu \nu }^a \widetilde{G}^{a,\mu \nu } A , \end{aligned}$$

from which we obtain the interaction vertices listed in Tables 9 and 10.

We match these effective vertices to the corresponding amplitudes induced by a quark loop, which couples to the $H$ and $A$ components of $X_0$ accordingly to Eq. (1) ($\kappa _{\scriptscriptstyle Htt,Att}= 1$), in the limit where this quark is infinitely massive. As a consequence, the effective couplings are fixed to the values

24 $$\begin{aligned} g_{\scriptscriptstyle Hgg} = -\frac{\alpha _s}{3 \pi v} \quad \mathrm {and} \quad g_{\scriptscriptstyle Agg} = \frac{\alpha _s}{2 \pi v}. \end{aligned}$$

Our effective theory is invariant under $\mathrm{SU}(3)_C$, so we can consistently add higher-order QCD corrections. Going to NLO, we match again the result from the effective theory to the corresponding case where the amplitude is induced by a heavy-quark loop. In the latter case, virtual corrections consist of two-loop diagrams; some of them appear explicitly in the effective theory as one-loop diagrams, while the other ones simply result in a correction to the value of the effective coupling. This correction can be computed by means of a low-energy theorem [28]; for the scalar we have

25 $$\begin{aligned} g_{\scriptscriptstyle Hgg}&= -\frac{\alpha _s}{3 \pi v} \Big ( 1 +\frac{11}{4}\, \frac{\alpha _s}{\pi } \, + \mathcal {O}\big (\alpha _s^2 \big ) \Big ), \end{aligned}$$

while in the pseudoscalar case the effective coupling

26 $$\begin{aligned} g_{\scriptscriptstyle Agg}&= \frac{\alpha _s}{2 \pi v} \, \end{aligned}$$

is exact to all orders in $\alpha _s$ [113]. Together with this finite contribution to the UV renormalisation of the effective couplings, we also need the UV polar terms that appear in $D=4-2 \epsilon$ dimensional regularisation. Such counterterms are simply obtained by plugging into Eq. (24) the known $\overline{\text {MS}}$ renormalisation of the strong coupling

27 $$\begin{aligned} \alpha _s \rightarrow \alpha _s \Big (1 - \frac{1}{\epsilon }\, \frac{\alpha _s}{2 \pi }\, b_0 + \mathcal {O}\big (\alpha _s^2 \big ) \Big ), \end{aligned}$$

where $b_0$ is the first coefficient of the QCD beta function

28 $$\begin{aligned} b_0 = \frac{11}{6}\, C_A - \frac{2}{3}\, T_F \, n_f. \end{aligned}$$

Therefore, the UV counterterms have structures analogous to the tree-level Feynman rules in Tables 9 and 10.

To complete our set of rules, in Tables 11 and 12 we report the $R_2$ counterterms [114, 115] of our effective theory, needed for the automatic computation of one-loop amplitudes with the OPP method [65]. The $R_2$ vertices for $GGH$ have already been published in [68] (with slightly different conventions), while the $R_2$ vertices for the $G \widetilde{G} A$ operator are presented here for the first time.

## Footnotes

1 The CP-odd case does not have the $ggggX_0$ vertex due to the antisymmetric nature of the interaction.

2 The definition is analogous to Eq. (4.1) in [18].
